# R3DG: Retrieve, Rank, and Reconstruction with Different Granularities for Multimodal Sentiment Analysis

**DOI:** 10.34133/research.0729

**Published:** 2025-07-02

**Authors:** Yan Zhuang, Yanru Zhang, Jiawen Deng, Fuji Ren

**Affiliations:** ^1^ College of Computer Science and Engineering, University of Electronic Science and Technology of China, Chengdu 611731, China.; ^2^ Shenzhen Institute for Advanced Study, University of Electronic Science and Technology of China, Shenzhen 518000, China.

## Abstract

Multimodal sentiment analysis (MSA) aims to assess emotional states by integrating information from text, audio, and video. However, the heterogeneous nature of these modalities presents substantial challenges for accurate sentiment prediction. Existing approaches typically align pairs of modalities using attention mechanisms or contrastive learning, which are computationally expensive. Additionally, they often rely on a single granularity of alignment, either by averaging features over all time steps or aligning features at each individual time step. These approaches overlook the fact that emotional expression can vary across individuals and contexts, requiring multiple granularities to capture emotion effectively. To address these challenges, we propose a novel framework, Retrieve, Rank, and Reconstruction with Different Granularities (R3DG). R3DG segments the audio and video modalities into multiple representations at varying granularities based on their temporal durations. It then selects the most relevant representations that align closely with the text modality. To preserve the original information, R3DG reconstructs the audio and video data using the selected representations. Finally, the fused audio, video, and text features are aligned and combined for sentiment prediction, reducing the need for multiple alignment steps. Extensive experiments on 5 benchmark MSA datasets demonstrate that R3DG outperforms existing methods and achieves substantial reductions in computational time. Code is available at https://github.com/YetZzzzzz/R3DG.

## Introduction

**Fig. 1.**
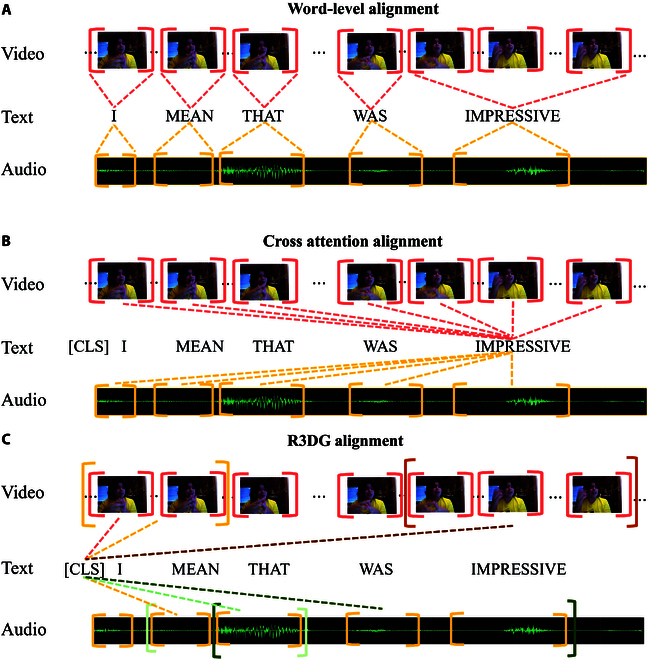
Illustration of different alignment strategies. (A) Illustration of word-level alignment. (B) Illustration of cross-modal attention alignment between each word in text (e.g., “impressive”) and each segment in video/audio. (C) The depiction of our R3DG. The different colored dashed lines represent varying levels of granularity, indicating the features involved in the fusion process. Larger brackets signify higher information content and broader integration scope. Matching colors between brackets and dashed lines indicate corresponding pairs where features from different modalities are aligned.

Multimodal sentiment analysis (MSA) aims to evaluate emotional states by integrating information from text, audio, and video modalities [1,2]. With the rapid advancements in deep learning and human–computer interaction, MSA has gained increasing attention. However, the heterogeneity between these modalities presents challenges: while texts provide rich global context [3–5], audio and video offer fragmented, continuous data. To address these challenges, most existing methods attempt to align pairs of modalities [6–8]. For example, LNLN [8] aligns audio and video with text to capture multimodal interactions, and MulT [9] aligns pairs of modalities at each time step, which can be seen in Fig. 1B.

While effective, these methods often use a fixed alignment granularity. Some align at a coarse-grained level, pooling representations over all time steps [10–12], while others use fine-grained, step-by-step alignment [9,13] . Both approaches overlook the variability of emotional expression across individuals and contexts, which requires multiple granularities to accurately capture emotional cues. For example, coarse-grained methods may miss subtle emotional cues like “head nod”, “frown”, or “high pitch”, especially in long videos. On the other hand, fine-grained alignment can lead to fragmented representations, where emotional events are divided into multiple time steps, creating data redundancy. Furthermore, these methods are computationally expensive due to the need for extensive attention-based alignment.

Additionally, some methods align modalities based on word-level synchronization [10,12], such as P2FA [14], as shown in Fig. 1A. This process synchronizes word occurrences in audio and video with the corresponding text. While these methods ensure consistent alignment of acoustic and visual features with text, they still suffer from the limitation of using a fixed granularity. Furthermore, many of these alignment-based methods require substantial computational resources. For example, pairwise cross-modal alignment requires multiple rounds of attention-based computations, while contrastive learning methods often involve time-consuming pre-training.

To address these challenges, we propose a novel framework called Retrieve, Rank, and Reconstruction with Different Granularities (R3DG). As illustrated in Fig. 1C, R3DG segments video and audio into representations at multiple granularities and selects those most similar to the “[CLS]” token of BERT textual representations [15]. To preserve the original information in the selected representations, R3DG reconstructs them and minimizes the loss between the reconstructed and original representations. Since the selected video and audio representations are highly similar to the textual representations, this step implicitly aligns them, eliminating the need for explicit alignment between these modalities and text. However, as there is no direct alignment between video and audio, we first align and fuse these representations before aligning, fusing, and predicting with the textual representation. This process minimizes the number of alignment steps, thereby reducing computational costs by limiting the need for attention-based alignment across the video, audio, and text modalities.

The key contributions of our work are as follows:

• We introduce the R3DG framework, which effectively aligns audio and video modalities with text for MSA.

• R3DG integrates representations of varying granularities from video and audio, enhancing its ability to capture emotional nuances across modalities.

• R3DG achieves state-of-the-art performance on multiple datasets while substantially reducing computational time.

## Results and Discussion

### Benchmarks

To comprehensively assess the applicability of R3DG and its performance across different alignment settings, we evaluate it on 5 datasets across 3 tasks. The first task is MSA, which involves both sentiment intensity assessment and sentiment categorization. For this task, we use the MOSI [1] and MOSEI [2] datasets, and evaluate the models in both unaligned and word-level aligned settings. The second task is multimodal emotion recognition (MER), which focuses on classifying emotions into 7 categories using the unaligned CHERMA [16] dataset. The final task is multimodal humor detection (MHD), which aims to detect humorous content using the word-level aligned UR-FUNNY [17] and MUStARD [18] datasets. Detailed descriptions of these datasets are provided in Materials and Methods.

For the MSA task, we evaluate performance using the following metrics: (a) MAE (mean absolute error) to quantify differences between predicted and actual values; (b) Corr (correlation) to assess the relationship between regression values and ground truth; (c) Acc-2 for binary classification accuracy; (d) Acc-7 for 7-class classification accuracy; and (e) F1 score for binary classification tasks. Notably, Acc-2 and F1 scores are reported using segmental notations such as “negative/non-negative” and “negative/positive”, respectively. “Non-negative” encompasses scores equal to or above zero, while “positive” specifically refers to scores greater than zero. For the MHD task, we report the Acc-2 results based on the UR-FUNNY and MUStARD datasets. In the MER task, we report the F1 score for each emotion category, as well as the averaged F1 score across all 7 classes.

### Baselines

To provide a thorough comparison, we selected a diverse set of baseline models and evaluated them alongside R3DG across multiple datasets. These baselines can be broadly grouped into 2 major categories: fusion-based methods and alignment-based methods, depending on their core strategies for handling multimodal data.

Fusion-based models focus on effectively integrating information from different modalities. These include methods that model modality interactions explicitly or apply low-rank fusion and mutual information maximization techniques. For instance, TFN [19] captures unimodal, bimodal, and trimodal interactions through a 3-way Cartesian product, while LMF [20] processes each modality separately before applying low-rank fusion. E-MIB [21] reduces noise by enforcing an information bottleneck on unimodal representations. Other models, such as MAG-BERT [22], utilize pre-trained BERT-based transformers [15] to fuse audio and video with text, and SELF-MM [23] jointly trains multimodal and unimodal tasks to generate self-supervised subtask labels. While methods like ICCN [24], SPT [25], and AGM [26] learn the relations between the modalities before fusing them.

Alignment-based models, on the other hand, emphasize bridging gaps between heterogeneous modalities. These can be further divided into 3 subcategories: (a) Representation decomposition methods separate features into modality-specific and modality-shared components. For example, MISA [27] and FDMER [28] separate representations into distinct subspaces, while models like DFT [29], PMR [30], LFMIM [16], and BCFN [31] use shared transformers to capture common information across modalities. (b) Contrastive learning methods align modalities by encouraging similar representations for semantically related inputs. HyCon [11] clusters cross-modal samples with the same label, MCL [10] calculates correlations based on label consistency, and CONFEDE [12] further disentangles representations into similarity and dissimilarity components. (c) Cross-modal attention methods directly model interactions between modalities using attention mechanisms. Examples include MulT [9], TETFN [32], LNLN [8], ALMT [33], MIL [34], and MSG [35]. In the approaches mentioned above, all models use single-level granularity alignment. For instance, MulT aligns modalities at the finest granularity, where each time step’s representation is aligned, while models like CONFEDE and MCL align modalities by averaging representations across all time steps and using a global representation.

### Leading performance of R3DG in the MSA task

In this section, we analyze the performance of R3DG and baseline models on the MSA task, which can be seen in Tables 1 and 2. For the MOSI dataset, in both the unaligned and word-level aligned settings, R3DG achieves the best results in “negative/positive” Acc-2 and F1 scores. In the unaligned setting, R3DG outperforms MulT, which aligns the finest-grained local representations at each timestep. This demonstrates the effectiveness of R3DG in leveraging multiple granularities. Additionally, R3DG achieves the highest Acc-7 score in this setting, indicating its ability to better capture sentiment intensity across various emotional levels. In the word-level alignment setting for MOSI, R3DG ranks first or second across all metrics, outperforming models like MCL, CONFEDE, and MulT, which use a single granularity for alignment. This confirms the advantage of using multiple granularities for modality alignment, even when word-level alignment is applied.

**Table 1.** The comparison with baselines on MOSI. The best performance is in boldface and the second best is underlined. The left side of “/” represents the “negative/non-negative” classification performance, while the right side represents the “negative/positive” classification performance for both Acc-2 and F1.

Models	MAE(↓)	Corr(↑)	Acc-2(↑)	F1(↑)	Acc-7(↑)
Unaligned settings					
TFN [19]	0.901	0.698	-/80.2	-/80.7	34.9
LMF [20]	0.917	0.695	-/82.5	-/82.4	33.2
SELF-MM [23]	**0.713**	0.798	84.00/85.98	**84.42**/85.95	-
TETFN [32]	0.717	**0.800**	84.05/86.10	83.83/86.10	-
ICCN [24]	0.860	0.710	-/83.0	-/83.0	39.0
MulT ^a^ [9]	0.889	0.686	-/81.1	-/81.0	39.1
ALMT [33]	0.752	0.768	82.75/84.91	82.94/85.01	42.37
LNLN [8]	0.751	0.778	81.24/84.25	81.79/84.61	44.56
SPT ^a^ [25]	-	-	-/81.2	-/81.3	-
MulT ^a^^,^^c^	0.774	0.793	83.24/85.52	83.12/85.48	43.00
SPT ^a^^,^^c^	0.836	0.772	83.82/85.82	83.70/85.76	41.40
R3DG ^a^	0.717	0.791	**84.11**/**86.59**	84.00/**86.55**	**46.06**
Word-level alignment settings					
MulT ^b^ [9]	0.871	0.698	-/83.0	-/82.8	40.0
MSG [35]	0.748	0.782	-/85.7	-/85.6	45.3
HyCon [11]	0.713	0.790	-/85.2	-/85.1	46.6
E-MIB [21]	0.711	0.798	-/85.3	-/85.3	46.6
DFT [29]	0.906	0.689	-/83.7	-/83.6	33.2
MISA [27]	0.783	0.761	81.8/83.4	81.7/83.6	42.3
MAG-BERT [22]	0.731	0.789	82.54/84.3	82.59/84.3	-
MCL [10]	**0.708**	0.793	-/85.5	-/85.4	**47.9**
CONFEDE [12]	0.742	0.784	84.2/85.5	84.1/85.5	42.3
SPT ^b^ [25]	-	-	-/82.8	-/82.9	-
MCL ^c^	0.709	0.795	83.8/85.5	83.7/85.5	**47.9**
CONFEDE ^c^	0.727	0.791	83.1/84.9	83.1/84.9	44.5
MulT ^b^^,^^c^	0.768	0.770	82.92/84.43	82.86/84.41	43.65
SPT ^b^^,^^c^	0.801	0.773	81.75/84.58	81.52/84.46	43.36
R3DG ^b^	0.709	**0.801**	**84.38**/**86.56**	**84.21**/**86.48**	46.86

^a^ Denotes the same method under different alignment settings.

^b^ Denotes the same method under different alignment settings.

^c^ Indicates that the models are replicated under the same condition..

**Table 2.** The comparison with baselines on MOSEI. The best performance is in boldface and the second best is underlined. The left side of “/” represents the “negative/non-negative” classification performance, while the right side represents the “negative/positive” classification performance for both Acc-2 and F1.

Models	MAE(↓)	Corr(↑)	Acc-2(↑)	F1(↑)	Acc-7(↑)
Unaligned settings					
TFN [19]	0.593	0.700	-/82.5	-/82.1	50.2
LMF [20]	0.623	0.677	-/82.0	-/82.1	48.0
SELF-MM [23]	**0.530**	0.765	82.81/85.17	82.53/85.30	-
TETFN [32]	0.551	0.748	**84.25**/85.18	84.18/85.27	-
ICCN [24]	0.565	0.713	-/84.2	-/84.2	51.6
MulT ^a^ [9]	0.591	0.694	-/81.6	-/81.6	50.7
ALMT [33]	0.542	0.752	83.99/85.62	**84.53**/85.69	52.18
LNLN [8]	0.572	0.735	83.61/84.14	84.02/84.53	50.66
SPT [25]	-	-	-/82.4	-/82.7	-
MulT ^a^^,^^c^	0.554	0.754	83.11/85.88	83.39/85.76	51.17
SPT ^a^^,^^c^	0.674	0.729	83.19/85.33	83.42/85.19	43.31
R3DG ^a^	0.537	**0.771**	83.49/**86.65**	83.85/**86.60**	**53.32**
Word-level alignment settings					
MulT ^b^ [9]	0.580	0.703	-/82.5	-/82.3	51.8
MSG [35]	0.583	0.787	-/85.4	-/85.4	52.8
HyCon [11]	0.601	0.776	-/85.4	-/85.6	52.8
E-MIB [21]	0.588	**0.790**	-/85.4	-/85.4	53.2
DFT [29]	0.564	0.740	-/85.3	-/85.2	52.3
MISA [27]	0.555	0.756	83.6/85.5	83.8/85.3	52.2
MAG-BERT [22]	0.539	0.753	**83.79**/85.23	83.74/85.08	-
MCL [10]	0.590	0.787	-/85.9	-/85.8	53.4
CONFEDE [12]	**0.522**	0.780	81.7/85.8	82.2/85.8	**54.9**
SPT [25]	-	-	-/82.6	-/82.8	-
MCL ^c^	0.564	0.752	82.0/85.6	82.5/85.6	52.8
CONFEDE ^c^	0.537	0.769	79.0/84.3	79.8/84.4	53.1
MulT ^b^^,^^c^	0.578	0.752	80.92/85.11	81.52/85.15	51.15
SPT ^b^^,^^c^	0.623	0.749	81.84/85.19	82.34/85.20	47.30
R3DG ^b^	0.542	0.757	83.6/**86.10**	**83.85**/**85.99**	53.4

^a^ Denotes the same method under different alignment settings.

^b^ Denotes the same method under different alignment settings.

^c^ Indicates that the models are replicated under the same condition..

For the MOSEI dataset, Table 2 illustrates that R3DG continues to outperform other models. In the unaligned setting, it achieves the best overall performance, surpassing existing models in Acc-7, Corr, “negative/positive” Acc-2, and F1. In the word-level alignment setting, R3DG also shows strong performance, surpassing replicated models like CONFEDE and MCL, which incorporate additional alignment through label information and contrastive learning. These results provide strong evidence of the effectiveness of R3DG in handling MSA across various settings.

### Superior performance of R3DG in the MER task

In this section, we compare the performance of R3DG and baseline models on the MER task, which can be seen in Table 3. R3DG achieves the second-best performance in the “Fear” and “Disgust” categories, while outperforming all other models in the remaining emotion categories. This strong performance can be attributed to the varying duration of different emotions. Single-granularity models often struggle to capture the diverse emotional cues accurately. However, R3DG’s use of multi-granularity representations allows it to cover a wider range of temporal selections, making it more adept at identifying various emotional cues. Overall, R3DG outperforms the current best models by 1.97% across all categories, demonstrating the advantage of using multi-granular representations to align diverse emotional cues and deliver more robust performance. In contrast, other alignment methods rely on a single granularity, limiting their ability to capture the full range of emotional categories effectively.

**Table 3.** The comparison with baselines on CHERMA in terms of F1-score. “Ha”, “Sa”, “Fe”, “An”, “Su”, “Di”, and “Ne” denote “Happy”, “Sadness”, “Fear”, “Angry”, “Surprise”, “Disgust”, and “Neutrality”, respectively. All models are evaluated under unaligned settings. The best performance is in boldface and the second best is underlined.

Models	Ha (↑)	Sa (↑)	Fe (↑)	An(↑)	Su (↑)	Di (↑)	Ne (↑)	Avg. (↑)
TFN [19]	74.91	75.56	66.15	74.41	66.29	43.34	65.60	68.37
LMF [20]	74.52	75.83	66.73	74.55	65.08	45.70	65.64	68.23
MulT [9]	76.18	76.88	67.36	74.85	68.18	46.96	65.26	69.24
PMR [30]	75.68	76.46	67.97	75.43	67.37	48.93	66.59	69.53
BCFN [31]	75.81	78.98	**82.16**	75.62	68.37	48.16	67.36	71.36
LFMIM [16]	76.60	77.83	69.44	75.32	69.83	**50.20**	68.24	70.54
R3DG	**81.38**	**81.15**	74.83	**77.74**	**70.30**	49.91	**69.64**	**73.33**

### Outstanding performance of R3DG in the MHD task

In this section, we compare the performance of R3DG and baseline models on the MHD task, which can be seen in Table 4. R3DG shows a marked performance advantage, with a 1.51% improvement on the UR-FUNNY dataset and a 4.41% improvement on the MUStARD dataset. Both datasets provide contextual information for each utterance, with an average context duration of about 15 s, while the target utterances typically span only around 5 s, leading to longer video content. This advantage highlights the limitations of methods that rely on single-granularity representations across the time dimension. Methods that use coarse-grained global representations often fail to capture short-term emotional fluctuations because they average over large portions of neutral content, while fine-grained representations may split emotional events across multiple representations, introducing new errors. In contrast, R3DG effectively integrates multi-granular information, selecting the most relevant aspects to improve performance.

**Table 4.** The comparison with baselines on UR-FUNNY and MUStARD, in terms of Acc-2. Models in parentheses indicates the textual features used. All models are evaluated under word-level alignment settings. The best performance is in boldface and the second best is underlined.

Models	UR-FUNNY (↑)	MUStARD (↑)
MISA(BERT) [27]	69.62	66.18
MISA(ALBERT) [27]	69.82	66.18
MAG(ALBERT) [22]	67.20	69.12
MAG(XLNet) [22]	72.43	76.47
AGM (BERT) [26]	65.97	-
FDMER (BERT) [28]	71.87	-
MIL (BERT) [34]	-	76.36
R3DG (BERT)	**73.94**	**80.88**

### Importance of the components of R3DG

Despite R3DG demonstrating strong performance across multiple datasets, it is important to further investigate whether R3DG truly utilizes information from the video and audio modalities, and which modality is more crucial. Additionally, we explore whether performance changes when similarity is considered without retaining sufficient original information. In this section, we conduct a detailed analysis to address these questions.

#### Importance of each modality

We first examine the importance of each modality across the datasets in Table 5. By systematically removing one modality at a time, we assess the impact on performance. For example, a model labeled “V+A” excludes the textual modality and uses only audio and video for fusion, along with their corresponding retrieval, ranking, reconstruction, and fusion components. The complete model, which includes all 3 modalities, is referred to as “V+A+T”. Table 5 shows that the removal of any modality leads to a performance drop across all datasets. Notably, excluding the textual modality causes the largest decline in performance, particularly on the MOSI and MOSEI datasets. This is partly because other modalities rely on the textual modality to select similar representations, and replacing it with an average representation loses important information. Additionally, the textual modality tends to dominate sentiment prediction in datasets like MOSI and MOSEI [36,37]. In contrast, the CHERMA dataset is less reliant on text, with audio and visual modalities playing a more marked role.

**Table 5.** Ablation studies for modules in R3DG with unaligned settings on MOSI, MOSEI and CHERMA datasets. The best performance is in boldface and the second best is underlined. “T”, “A”, and “V” denote the text, audio, and video modalities, respectively.

Configs	MOSI		MOSEI		CHERMA
	Acc-2(↑)	F1(↑)	Acc-2(↑)	F1(↑)	F1 (↑)
Role of each modality					
A+T	83.09/85.37	82.92/85.28	82.51/85.69	82.97/85.70	68.33
V+A	63.65/64.79	62.32/63.66	69.80/64.72	64.53/57.59	71.41
V+T	83.67/85.67	83.56/85.62	83.58/85.66	83.85/85.58	65.74
V+A+T	**84.11**/**86.59**	**84.00**/**86.55**	83.49/**86.65**	83.85/**86.60**	73.33
Role of retrieving					
${\text{Retri}}_{1}$	83.53/85.52	83.45/85.50	82.23/86.05	82.70/86.03	72.88
${\text{Retri}}_{\mathrm{all}}$	83.24/85.67	83.01/85.53	83.60/86.16	83.91/86.11	**73.38**
Role of ranking					
Random	83.09/85.52	82.90/85.42	83.07/85.77	83.37/85.67	72.96
${\mathrm{All}}_{\text{gran}}$	83.24/85.52	83.17/85.51	83.39/86.16	83.66/86.04	73.33
Role of reconstruction					
w/o $L_{\mathrm{re}}$	83.97/86.28	83.88/86.26	**83.92**/86.10	**84.18**/86.01	72.93

#### Importance of retrieval

Next, we analyze whether more granular representations lead to better performance. We compared coarse-grained global representations and fine-grained local representations. Specifically, we examined global representations obtained through max-pooling over the time dimension for all modalities, denoted as ${\text{Retri}}_{1}$, which aligns with methods like MAG-BERT [22]. We also explored using the finest-grained local representations by selecting the top 40 representations, referred to as ${\text{Retri}}_{\mathrm{all}}$. This is in contrast to models like MulT [9], which use a single granularity in unaligned settings. Our ablation experiments consistently show that finer-grained top-*k* representations lead to better performance than global representations, achieving state-of-the-art results on the CHERMA dataset. However, on the MOSI and MOSEI datasets, using a single granularity is suboptimal compared to integrating multiple granularities, highlighting the benefits of multi-granularity integration.

#### Importance of ranking

We then investigate whether the number of representations chosen for fusion impacts performance. Specifically, we examined 2 ranking strategies. In the first experiment, labeled “Random”, we randomly selected *k* representations from all granularity levels. In the second experiment, labeled ${\mathrm{All}}_{\text{gran}}$, we fused all representations across granularities without selection. Our results show that models using all representations across granularities perform better than those relying on random selection. However, they still underperform compared to R3DG selecting the most relevant top-*k* local representations. This finding emphasizes the importance of effectively selecting and prioritizing key local representations when integrating features from multiple granularities.

#### Importance of reconstruction

Finally, we evaluate the impact of excluding the reconstruction component, which attempts to retain the original information of selected representations. Our results indicate that omitting the reconstruction component yields performance results similar to those of the full R3DG model. This suggests that the top-*k* representations already capture a sufficient amount of information. Additionally, in datasets like MOSI and MOSEI, varying video lengths often lead to zero-padding during feature extraction, which may enhance performance. In contrast, datasets like CHERMA, which have more uniform feature values and consistent video durations, show a more noticeable performance drop when the reconstruction component is removed.

### Effectiveness of selected representations

Although Table 5 highlights the importance of each component of R3DG in its performance, there are still several aspects that need further investigation. For instance, while R3DG incorporates representations from different granularities of the video and audio modalities, questions remain about how many local representations from each granularity should be selected for alignment. Additionally, it is unclear where these selected representations are located within the original samples and whether there is any overlap of information between representations of different granularities. Furthermore, the specific contribution of the reconstruction component to R3DG’s overall performance also requires closer examination. This section delves into these questions in greater detail.

#### Effects of varying number of selected representation

**Fig. 2.**
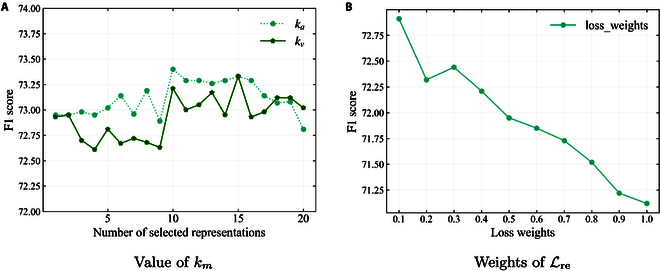
The performance of R3DG on CHERMA with (A) different values of $k_{m}$ in audio and video modalities, and (B) different weights of $L_{\mathrm{re}}$.

In this section, we investigate the effect of selecting varying numbers of local representations from the audio and video modalities on the CHERMA dataset, denoted by different values of $k_{m}$, where $m\in \left\{a,v\right\}$ represents the audio (*a*) and video (*v*) modality. The default configuration involves granularities of [5, 10, 15, 20], resulting in a total of 50 local representations. As shown in Fig. 2A, increasing the number of selected local representations substantially improves R3DG’s performance by providing more information. Specifically, for the audio modality, selecting 10 representations yields the highest average prediction accuracy, while for the video modality, selecting 15 representations results in the best performance. These findings demonstrate the effectiveness of selecting different numbers of local representations for different modalities and granularities.

#### Distributions of selected indices

In this section, we analyze the distribution of indices for the selected local representations to examine their locations and determine whether there is any overlap in the information they provide. Specifically, we visualize the frequency of selection and the average cosine similarity of indices across the unaligned MOSI and UR-FUNNY datasets. The analysis is conducted using the default settings with granularities of [5, 10, 15, 20], resulting in a total of 50 local representations across both the video and audio modalities.

**Fig. 3.**
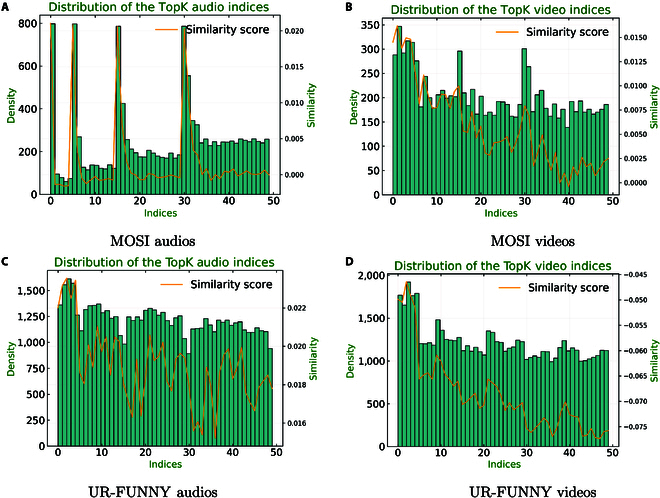
The distribution of the top-*k* indices in the unaligned MOSI audios (A), videos (B), and unaligned UR-FUNNY audios (C) and videos (D). The orange lines represent the averaged cosine similarities of the indices at different granularities.

As shown in Fig. 3, indices selected more frequently tend to exhibit higher cosine similarities. In particular, representations with more substantial temporal coverage—such as the top 5—are selected more often and have higher average cosine similarities. On the other hand, representations with shorter time spans, located toward the end of the axis, are selected less frequently and show lower similarity. In the MOSI dataset, for example, the most frequently selected indices in the audio modality correspond to the initial representations of each granularity, such as indices 0, 5, 15, and 30. These indices are selected more than 3 times as often as others and exhibit the highest cosine similarities, suggesting that there is some overlap in the information they provide.

In the video modality, across both datasets, we observe that while the selection frequency of indices varies, their cosine similarity tends to decrease as the granularity becomes finer. This indicates that finer granularities capture more specific details, but these details may diverge from the broader context, particularly in longer videos, where padding is often applied to maintain a consistent input length. This padding can result in sparse, less informative representations at the end of the video.

#### Effects of different weights of reconstruction

In this section, we examine the contribution of the reconstruction to R3DG’s overall performance through varying weights of $L_{\mathrm{re}}$. As shown in Fig. 2B, increasing the weight values results in fluctuating declines in performance. The best performance is achieved with a weight value of 0.1, while performance noticeably worsens when the weight reaches 1.0. On average, lower weight values tend to correlate with better relative performance, while higher weight values generally lead to poorer results.

### Superior efficiency of R3DG

Existing alignment methods typically rely on single-granularity approaches, but they often require multiple attention-based alignments, each with a time complexity of $O\left(N^{2}\right)$, leading to substantial computational cost. For example, the MulT model aligns representations across all time steps, resulting in a time complexity of $O\left(\left(M_{L}M_{V}+M_{L}M_{A}+M_{A}M_{V}\right)N^{2}\right)$, where $M_{m}$ is the time length of modality *m*, $m\in \left\{L,A,V\right\}$. Similarly, models like CONFEDE not only include the above alignment time complexity but also require pre-training on various datasets, further increasing their computational overhead.

In contrast, R3DG reduces this computational burden by performing alignment just twice: once between the video and audio modalities, and then aligning the fused representations with the text modality. This results in a total time complexity of only $O\left(2N^{2}\right)$, much lower than the time required by MulT and CONFEDE.

To provide a more intuitive comparison, we evaluate the computational complexity of R3DG relative to several baseline models, considering both time and space requirements. For space complexity, we analyze the number of parameters in each model. In terms of time complexity, we compare the total runtime of each model over 100 epochs under the same experimental settings, including a batch size of 128. As shown in Table 6, R3DG has a slightly higher parameter count than the other models. However, it demonstrates strong runtime efficiency. For instance, in word-level alignment settings, R3DG completes the task in just 384 s. Among all models, only SPT has a comparable runtime at 392 s, while others require at least 500 s. In unaligned settings, R3DG finishes in 857 s—slightly longer than SELF-MM but substantially faster than other models, which require over 1,500 s. This efficiency is due to R3DG’s design, which selects a subset of local representations for fusion before applying cross-attention for alignment. By reducing the number of attention computations, R3DG effectively lowers the overall computational cost. While SELF-MM is slightly faster in unaligned settings and also performs well, R3DG achieves better overall results, particularly on datasets like MOSEI. These findings highlight R3DG’s ability to balance computational efficiency with strong performance, making it a compelling choice for MSA.

**Table 6.** Comparison of parameters and running time for different models on the MOSI dataset

Models	# Params (↓)	Running time (↓)
MISA (Aligned) [27]	110,620,273	535 s
ConFEDE (Unaligned) [12]	246,998,404	6,018 s
MulT (Unaligned) [9]	110,862,889	2,205 s
MulT (Aligned) [9]	110,874,409	558 s
SELF-MM (Unaligned) [23]	109,641,572	765 s
SPT (Unaligned) [25]	109,466,497	1,522 s
SPT (Aligned) [25]	109,472,641	392 s
R3DG (Unaligned)	112,660,865	857 s
R3DG (Aligned)	112,697,729	384 s

### Case visualization

**Fig. 4.**
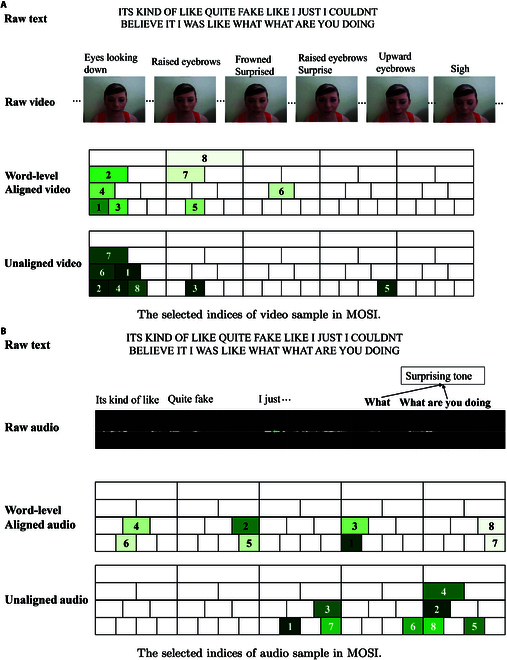
An example in MOSI. Given the text “ITS KIND OF LIKE QUITE FAKE LIKE I JUST I COULDNT BELIEVE IT I WAS LIKE WHAT WHAT ARE YOU DOING” and the corresponding video (A) and audio (B) files with both word-level aligned and unaligned settings, our R3DG framework identifies the most relevant segments. In the visual representation, boxes are labeled with numbers (e.g., 1, 2) indicating the top-*k* relevant parts, where darker colors signify higher similarity scores.

To better illustrate the effectiveness of R3DG, we present a detailed analysis using an example from the MOSI dataset, comparing the selected representations under both word-level aligned and unaligned settings, as shown in Fig. 4. The aim is to demonstrate how the selected local representations are distributed for the example sentence: “ITS KIND OF LIKE QUITE FAKE LIKE I JUST I COULDNT BELIEVE IT I WAS LIKE WHAT WHAT ARE YOU DOING.” Each box in the visualization represents a local representation, with its length indicating the granularity of the feature. Overlapping boxes within a column signify information overlap, while the numbers (e.g., 1, 2) indicate the most and second-most similar local representations for each modality.

We observe distinct differences in the top 8 similarity scores for both audio and video modalities between the aligned and unaligned settings. In the unaligned settings, similarity scores are generally higher, suggesting that word-level alignment might smooth out subtle variations and potentially lead to the loss of key information. In the video modality, while there is marked information overlap in both settings, the selection of features differs. In the word-level alignment setting, the selected representations are concentrated in the first 40% of the timeline. Conversely, in the unaligned setting, more emphasis is placed on the first 20% of the timeline, focusing on finer-grained features such as “upward eyebrows”, which are not prioritized in the aligned setting.

The differences in the audio modality between the 2 settings are even more pronounced. In the word-level alignment setting, features are selected throughout most of the audio timeline, corresponding to changes in intonation and emphasized speech. However, in the unaligned setting, there is greater focus on the final segment of the audio, capturing a noticeable tone of surprise, which aligns more closely with the sentiment expressed in the text. Other parts of the audio timeline are given less attention in this setting.

This example demonstrates R3DG’s high ability to identify multimodal similarities at various granularities and showcases its robustness in handling both word-level alignment and unaligned scenarios.

### Limitations of R3DG

While R3DG exhibits competitive overall performance across multiple datasets, certain limitations are observed in specific scenarios that require fine-grained sentiment recognition. For instance, on the MOSI and MOSEI datasets, R3DG performs slightly lower than some state-of-the-art models on fine-grained metrics such as MAE, Corr, and Acc-7. Similarly, on the CHERMA dataset, R3DG shows reduced effectiveness in distinguishing subtle emotions like “Fear” and “Disgust”, though it handles more salient emotions such as “Sadness” and “Happy” well. These cases reveal a limitation in R3DG’s current ability to consistently capture nuanced sentiment distinctions.

This limitation may be partly attributed to redundancy in the representation selection process. As illustrated in Fig. 4A, highly similar representations often cluster within narrow temporal segments, which can limit the diversity of the information captured. Such concentration may restrict the R3DG’s ability to attend to temporally distributed cues that are crucial for identifying subtle emotional variations.

To address this, a potential enhancement lies in encouraging temporal diversity during representation selection. By retaining the most informative representations while reducing redundancy—especially among those with high similarity in close time spans—R3DG can be guided to leverage a broader context, which may improve its performance in fine-grained classification tasks.

Overall, despite these task-specific limitations, R3DG remains a strong and generalizable framework for MSA. Future work will focus on refining the representation learning mechanism to improve its sensitivity to nuanced affective expressions.

## Conclusion

This paper introduces the R3DG framework for MSA. By effectively aligning audio and video modalities to the text modality with varying granularities, R3DG not only reduces computational complexity but also enhances the ability to capture nuanced emotional fluctuations across different modalities. The ability to segment and select the most relevant audio and video representations, while preserving critical information through reconstruction, allows for more accurate and efficient sentiment prediction. Experimental results demonstrate that R3DG achieves state-of-the-art performance in multiple multimodal tasks, including sentiment analysis, emotion recognition, and humor detection, outperforming existing methods. Ablation studies further confirm R3DG’s superiority, highlighting its robust performance despite the reduced computational cost. Looking ahead, future work will focus on automating the selection of modality importance and granularity, further enhancing R3DG’s adaptability to diverse real-world applications.

## Materials and Methods

### Datasets and evaluation metrics

In order to comprehensively evaluate the performance of R3DG, 5 datasets are selected, including MOSI [1], MOSEI [2], UR-FUNNY [17], MUStARD [18], and CHERMA [16]. These datasets are categorized based on their respective task objectives: MOSI and MOSEI for MSA, UR-FUNNY and MUStARD for MHD, and CHERMA for MER. Specifically, MSA aims to predict sentiment polarity and categorize it based on predefined ranges. MOSI comprises 2,198 utterances extracted from YouTube videos, each averaging 4.2 s in duration, with regression labels ranging from −3 (strong negative) to 3 (strong positive). MOSEI is an expanded version of MOSI, consisting of 23,453 utterances with consistent label information. R3DG in unaligned settings aligns with previous studies [9], while word-level alignment settings follow similar methodologies [10,11]. MHD focuses on binary classification to determine whether content contains humor. This task involves the UR-FUNNY [17] and MUStARD [18] datasets, which uniquely include contextual information. UR-FUNNY consists of 9,588 TED utterances, with durations averaging 14.7 s for contexts and 4.58 s for punchlines. MUStARD comprises 690 utterances from TV shows, with context durations averaging 13.95 and 5.22 s, respectively. MER addresses multi-class emotion recognition, evaluating emotional categories using the CHERMA [16] dataset. This dataset includes 7 emotions: “Happy”, “Sadness”, “Fear”, “Angry”, “Surprise”, “Disgust”, and “Neutrality”, sourced from 28,717 Chinese utterances across various media websites.

### Task definition

MSA aims to utilize diverse, heterogeneous data modalities to determine the category or polarity of emotions. These modalities typically encompass text, video, and audio [1,38]. We focus exclusively on tasks involving these 3 modalities. To facilitate analysis, we represent the features of each modality as $U_{m}\in {\mathbb{R}}^{T_{m}\times d_{m}}$, where $m\in \left\{t,a,v\right\}$ denotes the modality, and $d_{m}$ and $T_{m}$ represent the respective dimensionality and sequence length. Given *i*th utterance with representation $\left\{U_{t,i},U_{a,i},U_{v,i}\right\}$, MSA predicts its emotion intensity or category ${\hat{y}}_{i}$. For MSA task with the MOSI and MOSEI datasets, ${\hat{y}}_{i}$ is the regression values, while for the MHD task and MER task, the ${\hat{y}}_{i}$ is the logits for each emotion category.

#### Model structure

##### Retrieve and rank module

**Fig. 5.**
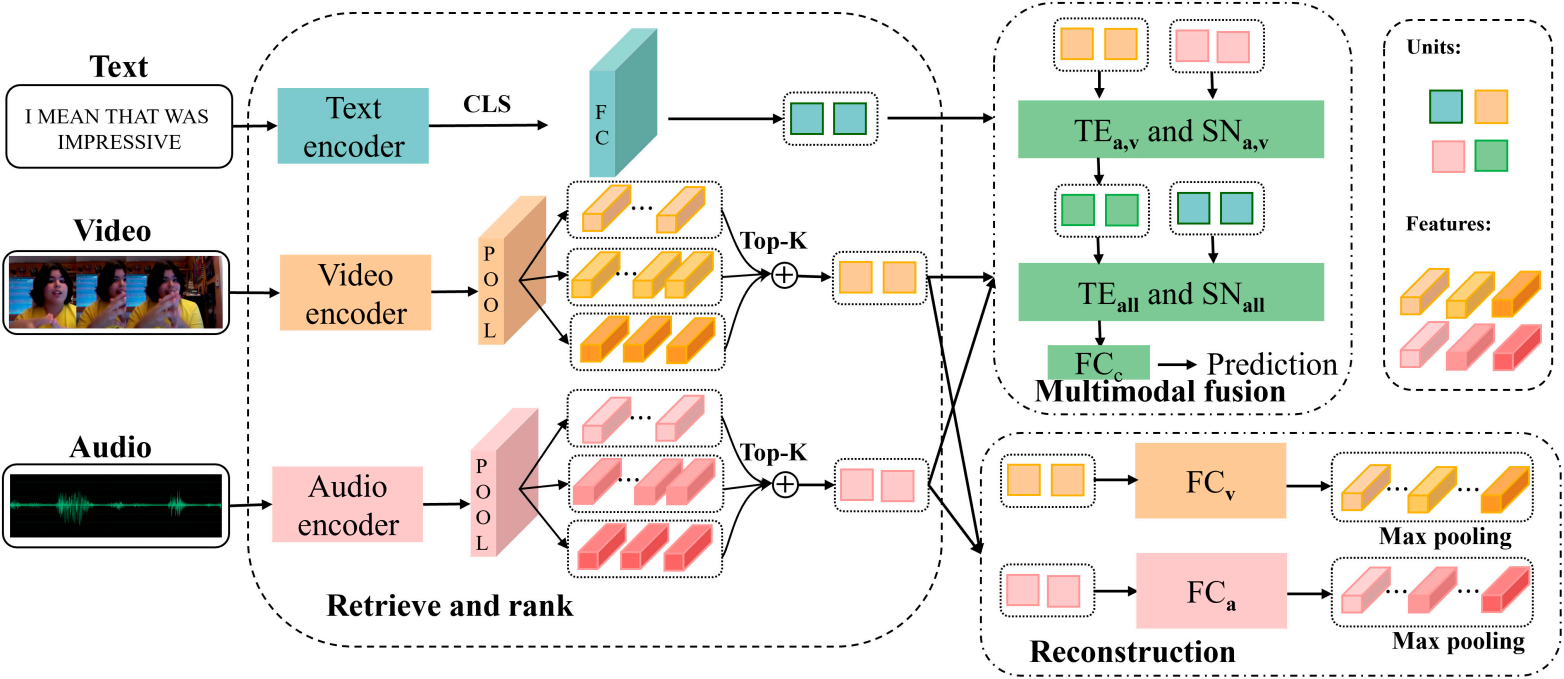
Illustration of our R3DG framework. It consists of 3 submodules. The retrieve and rank module acquires representations from each modality, employing adaptive pooling to capture various granularities of video and audio modalities. It ranks and selects the top-*k* representations most similar to textual features for fusion. The reconstruction module reconstructs the original audio and video representations through the fused local audio and video representations. Lastly, the multimodal fusion module integrates the extracted features from each modality for prediction. Different colors of cubes in the video and audio modalities denote varying granularities of local representations.

**Fig. 6.**
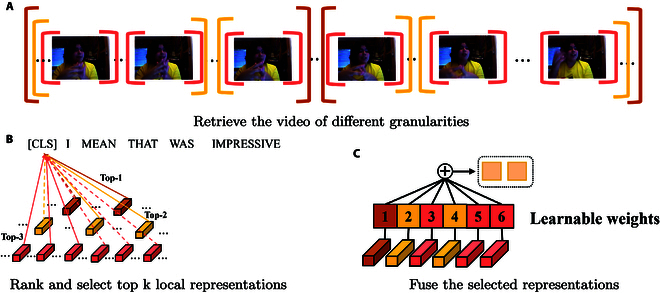
The simplified procedure of the retrieve and rank module. (A) The use of adaptive max pooling to extract local representations at different granularities. Each color represents a distinct granularity, with the length of the brackets indicating the stride and duration of temporal segments—larger brackets correspond to larger strides and encompass more information. (B) The process of calculating the similarity between each local representation and the textual representation, followed by ranking and selecting the top-*k* most similar representations. Here, *k* equals to 6. (C) The fusion of these top-*k* local representations, where their fusion weights are trainable parameters.

As shown in Fig. 5, the R3DG framework consists of three submodules: retrieve and rank module, reconstruction module and multimodal fusion module. Due to differences in sampling methods across modalities and the large volume of data in video and audio modalities, as well as substantial data overlap, the retrieve and rank module is designed to segment audio and video data into distinct representations at various granularities and assess their similarity to textual features. It subsequently ranks and selects the top several performing representations for fusion, employing learnable parameters to optimize integration across modalities, as shown in Fig. 6. This approach ensures effective alignment and enhances the coherence between modalities.

Specifically, for utterances with representations $\left\{U_{t},U_{a},U_{v}\right\}$, we first extract the unimodal representations of each modality. To be consistent with prior research [9–11], we adapt the similar methods to get these representations.

For text representations, we use the BERT [15] as the text encoder and choose the [CLS] token of the last layer as the text representations, then a fully connected layer is adapted to reduce the dimension and get the final representations. The above process can be seen in the following equation:

$${\hat{X}}_{t}=\text{BERT}\left(U_{t}\right)W_{t}^{⊤}+b_{t},$$ (1)

where ${\hat{X}}_{t}\in {\mathbb{R}}^{h_{t}}$, $h_{t}$ is the hidden dimension of the text modality, and $W_{t}\in {\mathbb{R}}^{h_{t}\times d_{t}}$ and $b_{t}\in {\mathbb{R}}^{h_{t}}$ are learnable parameters in the fully connected layer.

For audio and video representations, we extract the local representations of different granularities. Specifically, the temporal convolutional networks (Conv1D) and transformer encoder are firstly adapted to make sure that each element of the input sequences tends to the neighbor elements and the whole sequence, as the following equation shows:

$${\hat{X}}_{m}=\mathrm{TE}\left(\text{Conv}1D\left(U_{m}\right)\right),$$ (2)

where $m\in \left\{a,\;v\right\}$ denotes the modality, and the TE is the transformer encoder comprising a series of *N* layers, each with self-attention, normalization, and fully connected sublayers. For the input representation $U_{m}$, the query, key, and value are set as $Q,K,V=U_{m}$ in the self-attention layer:

$$Z_{m}^{\;}=\text{SELF ATTENTION}\left(U_{m}^{\;}\right)$$ (3)

$$=\text{softmax}\left(\frac{Q_{m}K_{m}^{⊤}}{\sqrt{d_{k}}}\right)V_{m}$$ (4)

$$=\text{softmax}\left(\frac{U_{m}W_{Q_{m}}W_{V_{m}}^{⊤}U_{m}^{⊤}}{\sqrt{d_{k}}}\right)U_{m}W_{V_{m}},$$ (5)

where ${\hat{X}}_{m}\in {\mathbb{R}}^{T_{m}\times h_{m}}$, and $h_{m}$ is the hidden dimension of the modality $m,m\in \left\{a,\;v\right\}$.

To retrieve the appropriate representations of the audio and video modalities, we use the AdaptiveMaxPool1d function to obtain the different granularities of the representations. Specifically, for each granularity, we obtain the local representations by setting the different number to the stride and kernels. Suppose we use s different granularities, and each has $g_{m}^{j},j\in \left\{1,\;2,\ldots ,\;s\right\}$ local representations, the *j*th output of modality *m* is obtained by:

$$L_{m}^{j}=\text{AdaptiveMaxPool}1d_{g_{m}^{j}}\left({\hat{X}}_{m}\right),$$ (6)

where $L_{m}^{j}\in {\mathbb{R}}^{g_{m}^{j}\times h_{m}}$ denotes the local representations with the granularity $g_{m}^{j}$ of the modality *m*. Thus, we obtain $S_{m}=\sum_{j=1}^{s}g_{m}^{j}$ local representations. Besides, we also extract the global representation by maxpooling function using all time steps for reconstruction, thus the $g_{m}^{j}=1$. Note that the global representation is only used for the reconstruction module, not for other modules, which means that the global representation will not be retrieved, ranked, and fused. Here, we use $G_{m}\in {\mathbb{R}}^{h_{m}},m\in \left\{a,\;v\right\}$ to denote it.

All local representations are stacked together for further ranking:

$$L_{m}^{\mathrm{all}}=\text{Concat}\left(L_{m}^{1},\ldots ,\;L_{m}^{s}\right),$$ (7)

where $L_{m}^{\mathrm{all}}\in {\mathbb{R}}^{S_{m}\times h_{m}}.$

After getting the local representations of different granularities of the audio and video modalities, we rank these representations based on their similarities with the text representations. Specifically, we choose the top $k_{m}$ local representations of modality *m*, and use the learnable weights to fuse them. Note that the learnable weights are learnable tensors, denoted as $W_{m},m\in \left\{a,\;v\right\}$.

$$\begin{array}{c} F_{m}={\text{TopK}}_{k_{m}}\left(\mathrm{Sim}\left(L_{m}^{\mathrm{all}},\;{\hat{X}}_{t}\right)\right)\text{Softmax}\left(W_{m}^{⊤}\right), \end{array}$$ (8)

where Sim denotes the cosine similarity, and $k_{m}$ denotes the number of the selected local representations of the modality *m*. Here, $W_{m}\in {\mathbb{R}}^{k_{m}}$, and TopK denotes the function to choose the top $k_{m}$ most similar local representations and concatenate them, thus $F_{m}\in {\mathbb{R}}^{h_{m}}$.

##### Reconstruction module

The varying lengths of videos and audios introduce challenges in selecting local representations. Focusing excessively on finer-grained features can overlook global information, posing potential issues. Moreover, due to granularity differences, selected local representations inevitably exhibit information overlaps. The reconstruction module aims to distribute these potentially overlapped local representations more uniformly across the entire input sequence and duration, rather than being confined to specific token positions. It not only maintains granularity and unique information in these local representations but also ensures relative global coverage.

Specifically, we use the fully connected layer for reconstruction and use the mean squared error (MSE) to reduce the gap between the fused local representations and global representations:

$$L_{\mathrm{re}}=\mathrm{MSE}\left(F_{m}{W_{m}^{r}}^{⊤}+b_{m}^{r},\;G_{m}\right),$$ (9)

where $W_{m}^{r}\in {\mathbb{R}}^{h_{m}\times h_{m}}$ and $b_{m}^{r}\in {\mathbb{R}}^{h_{m}}$ are trainable parameters in the fully connected layer, and $m\in \left\{a,\;v\right\}$ denotes the modality.

##### Multimodal fusion module

In the retrieve and rank module, we select several local representations from video and audio modalities that exhibit the highest similarity to textual representations. Although this process effectively aligns textual representations with video and audio modalities, it lacks the direct alignment between video and audio. Therefore, in this module, we decompose multimodal fusion into 2 stages: first, integrating video and audio modalities, followed by fusing the combined result with textual modality. Specifically, we employ transformer encoder layers and linear layers for integration.

Specifically, the fused local representations of audio and video modalities, $F_{a}$ and $F_{v}$, are stacked and feed into transformer encoder layer and sequential network subsequently:

$$\begin{array}{c} {\text{Fuse}}_{a,v}={\mathrm{SN}}_{a,v}\left(\text{Concat}\left({\mathrm{TE}}_{a,v}\left(\text{Stack}\left(F_{a},\;F_{v}\right)\right)\right)\right). \end{array}$$ (10)

Then, the aligned and fused representations ${\text{Fuse}}_{a,v}$ are aligned and fused with the text representations, which can be formulated as:

$$\begin{array}{c} {\text{Fuse}}_{\mathrm{all}}={\mathrm{SN}}_{\mathrm{all}}\left(\text{Concat}\left({\mathrm{TE}}_{\mathrm{all}}\left(\text{Stack}\left({\hat{X}}_{t},\;{\text{Fuse}}_{a,v}\right)\right)\right)\right). \end{array}$$ (11)

The fused representations of all 3 modalities are finally fed into a fully connected layer for prediction.

$$\hat{y}={\mathrm{FC}}_{c}\left({\text{Fuse}}_{\mathrm{all}}\right),$$ (12)

where ${\mathrm{TE}}_{a,v}$ and ${\mathrm{TE}}_{\mathrm{all}}$ use the cross attention to align different modalities by setting the query input layer $Q=Z_{k}$, and the keys *K* and values *V* to $Z_{l}$ given the 2 input $Z_{k}$ and $Z_{l}$:

$$Z_{k,l}=\text{CROSS}\;\text{ATTENTION}\left(Z_{k},\;Z_{l}\right)$$ (13)

$$=\text{softmax}\left(\frac{Q_{Z_{k}}K_{Z_{l}}^{⊤}}{\sqrt{d_{k}}}\right)V_{Z_{l}}$$ (14)

$$=\text{softmax}\left(\frac{Z_{k}W_{Q_{Z_{k}}}W_{V_{Z_{l}}}^{⊤}{\left(Z_{l}\right)}^{⊤}}{\sqrt{d_{k}}}\right)Z_{l}W_{V_{Z_{l}}},$$ (15)

where ${\mathrm{SN}}_{a,v}$ and ${\mathrm{SN}}_{\mathrm{all}}$ are sequential networks of a linear layer, a dropout layer, and a ReLU activation layer.

##### Optimization object

In this section, the optimization objectives of our R3DG framework is introduced, which consists of reconstruction loss $L_{\mathrm{re}}$ and task-specific losses $L_{\text{task}}$. The $L_{\mathrm{re}}$, as defined by Eq. 9, encompasses the MSE between the reconstructed part of fused local representations and the maxpooling of the entire input sequence. Given that MSA involves both classification and regression tasks, we employ different $L_{\text{task}}$ accordingly.

For classification tasks, we utilize cross-entropy loss as

$$L_{\mathrm{cla}}=\frac{1}{M}\sum_{i=1}^{M}-y_{i}\text{log}\left({\hat{y}}_{i}\right).$$ (16)

For regression tasks, we opt for L1 loss:

$$L_{\mathrm{reg}}=\frac{1}{M}\sum_{i=1}^{M}\left|y_{i}-{\hat{y}}_{i}\right|,$$ (17)

where ${\hat{y}}_{i}$ is the predicted value, $y_{i}$ is the true label, and *M* is the number of all samples.

To strike a balance between reconstruction and task losses, we adopt a weighted approach, defined as:

$$L=L_{\text{task}}+\alpha L_{\mathrm{re}},$$ (18)

where α is a hyperparameter.

### Implementation details

In the aforementioned 5 datasets, UR-FUNNY and MUStARD employ word-level alignment, CHERMA remains unaligned, while MOSI and MOSEI incorporate both unaligned and word-level aligned data. The feature extraction methods for aligned and unaligned data are identical, differing only in the additional postprocessing steps. Therefore, we do not distinguish between these modalities in the current context. Word-level alignment involves manually averaging the occurrence times of words across speech and audio features to obtain synchronized alignments [39]. Specifically, the forced alignment model is utilized to extract word timings from both punch lines and contexts [14]. Subsequently, acoustic and visual features for the entire video are captured, and each word’s timing dictates the extraction of relevant segments of these features. These segmented feature arrays are separately averaged across the temporal dimension, resulting in acoustic and visual feature vectors for each word.

For the text modality, except for CHERMA where we utilize the official Chinese BERT embeddings [40], all other datasets maintain consistency with previous work [11,12,27,41] by employing the final layer’s [CLS] token from BERT-base [15,42] for representation. Regarding the audio modality, we utilize COVAREP [43], an acoustic analysis toolkit, to extract features including Mel-frequency cepstral coefficients, pitch, and voiced/unvoiced segmentation features for MOSI, MOSEI, UR-FUNNY, and MUStARD datasets. For CHERMA, pre-trained wav2vec [44] is employed for feature extraction. In the visual modality, we employ Facet for extracting facial expression-related information for MOSI and MOSEI, and OpenFace [45,46] for UR-FUNNY and MUStARD datasets. For CHERMA, pre-trained ResNet18 [47] is utilized. It is noteworthy that the UR-FUNNY and MUStARD datasets include additional contextual information. Following the prior studies [39], we concatenated this contextual data with the original utterances before feeding them into modality specific networks for representation.

In our experiments, we define several key parameters. The learning rate is denoted by *lr*, the batch size by *bsz*, and the weight of the reconstruction loss by α. The hidden dimensionalities of the audio, video, and text modalities are represented by $h_{m},m\in \left\{t,\;a,\;v\right\}$. Parameters $k_{a}$ and $k_{v}$ specify the number of most similar local representations selected for the audio and video modalities, respectively. *N* represents the number of transformer encoder layers, while $g_{a}$ and $g_{v}$ define the granularity settings for the audio and video modalities.

All experiments were conducted using the PyTorch framework on a GTX 3090 GPU with CUDA 11.5 and Torch version 1.12.1. The AdamW optimizer [48] was used for model training. Hyperparameter tuning was performed via grid search to identify the optimal configuration that minimizes validation loss across all tasks. Given the diversity of the datasets, distinct hyperparameters were required for each. We explored a range of hyperparameter values, including batch sizes from {64, 96, 128, 168, 192}, learning rates from {8e−6, 9e−6, 1e−5, 2e−5, 3e−5, 4e−5, 5e−5}, reconstruction loss weight α from {0.05, 0.1, 0.15, 0.2, 0.25, 0.3}, hidden dimensions $h_{m},m\in \left\{t,a,v\right\}$ from {64, 96, 128, 160, 192}, transformer encoder layers *N* from {3, 4, 5, 6}, and selected top $k_{a}$ and $k_{v}$ representations for the audio and video modalities from {8, 9, 10, 11, 12, 13, 14, 15}. The granularity values $g_{a}$ and $g_{v}$ for audio and video were chosen from {[5, 10, 15, 20], [10, 20, 30, 40]}.

## Data Availability

All datasets used in this paper are publicly available. Specifically, the MOSI and MOSEI datasets are available at https://github.com/WasifurRahman/BERT_multimodal_transformer. The UR-FUNNY and MUStARD datasets are available at https://github.com/matalvepu/HKT/tree/main/dataset, and the CHERMA dataset is available at https://github.com/sunjunaimer/LFMIM.
